# Correspondence: On the nature of strong piezoelectricity in graphene on SiO_2_

**DOI:** 10.1038/ncomms11570

**Published:** 2016-05-17

**Authors:** Christoph Stampfer, Sven Reichardt

**Affiliations:** 1 JARA-FIT and 2nd Institute of Physics, RWTH Aachen University, 52074 Aachen, Germany; 2 Peter Grünberg Institute (PGI-9), Forschungszentrum Jülich, 52425 Jülich, Germany; 3 Physics and Material Science Research Unit, Université du Luxembourg, 1511 Luxembourg, Luxembourg

Spatially resolved Raman spectroscopy and piezoresponse force microscopy are very interesting and useful tools for investigating properties of graphene and other two-dimensional materials. In a recent article published in *Nature Communications*, da Cunha Rodrigues *et al*.^1^ used both methods to investigate single-layer graphene deposited on SiO_2_ grating substrates. Interestingly, the authors report on strong piezoelectricity and on high in-plane strain values of 3–5% in the supported graphene regions. It is argued that the in-plane strain originates from the strong interaction of the carbon atoms with the oxygen atoms of the SiO_2_ substrate. Their finding of high in-plane strain is crucial, as it is considered to be of the same origin as the observed strong piezoelectricity in graphene on SiO_2_. Unfortunately however, a major correction is needed. The strain values reported by da Cunha Rodrigues *et al*. appear to be more than a factor 50 too large, that is, the actual strain in their investigated samples is only on the order of 0.06–0.10% or lower.

To extract the amount of strain in their samples, the authors employ confocal Raman spectroscopy, which has been shown to be a reliable tool for this purpose^2^,^3^,^4^,^5^. The reported values of the Raman G band frequencies are within a reasonable and expected range for graphene supported by SiO_2_ (refs 6, 7, 8), with the average frequency on the supported region around 1,587 cm^−1^ and a frequency spread on the order of 1 cm^−1^. To convert these Raman shifts into strain values, the authors assume that the strain is of uniaxial nature and use a Grüneisen parameter of Δ*ω*_G_/Δ*ɛ*=−49.3 cm^−1^=−0.493 cm^−1^/% (see page 6 in Supplementary Note 1 of da Cunha Rodrigues *et al*.^1^). Correspondingly they extract strain values in the range of 3–5% in the supported region. However, the Grüneisen parameter taken from Bisset *et al*.^9^ (ref. 3 in Supplementary Note 1 of da Cunha Rodrigues *et al*.^1^) should read Δ*ω*_G_/Δ*ɛ*=−49.3 cm^−1^/% (see text in Bisset *et al*.^9^). Please note that in Table 1 of Bisset *et al*.^9^, there unfortunately is a mistake in the units: cm^−1^ should be replaced by cm^−1^/%; the units are however fine in the text. By using the correct units, this leads to strain values which are exactly a factor 100 smaller than what has been claimed by the authors.

Moreover, using more established Grüneisen parameters for uniaxial strain, which range from −21.3 to −23.5 cm^−1^/% (refs 2, 10) and are in good agreement with the results of a first principles calculation^2^, results in strain values of 0.06–0.10%, instead of the claimed 3–5%. Furthermore, it is not clear whether the nature of the strain is uniaxial. Assuming biaxial strain instead, the extracted strain values are reduced by a further factor of 3 if one uses a Grüneisen parameter of −69.1 cm^−1^/%, as previously measured for biaxial strain^5^, which would result in an in-plane strain of 0.02–0.03% only.

In view of these corrected, smaller values of in-plane strain, we think the authors should reconsider their interpretation of the observed strong piezoelectricity. The authors themselves assume the origin of their reported high in-plane strain values to be the very same as the one of the strong piezoelectricity. The latter is attributed to non-zero net dipole moments in the system due to the chemical interaction between carbon and oxygen atoms, while at the same time the C–O bonds are said to be the origin of the high in-plane strain values^1^.

However, except for the claimed very high in-plane strain, the paper offers no clear motivation of why carbon–oxygen bonds should form at the interface between graphene and the SiO_2_ substrate. While the authors do cite two theoretical studies^11^,^12^ to support their claim of strong covalent C–O bonds, these studies investigate the highly idealized geometry of graphene on crystalline, α-quartz SiO_2_. As also commented upon in these studies, the experimental situation significantly differs from this scenario, since the commonly used commercially available Si/SiO_2_ wafers, as also used in the experiment by da Cunha Rodrigues *et al*.^1^, feature amorphous SiO_2_, on which graphene forms much weaker bonds. A more careful theoretical calculation^13^, while still assuming crystalline SiO_2_, used non-cleaved, fully oxygen-terminated surfaces and found only weak bonds between carbon and oxygen atoms and only small dipole moments induced in the interfacial region. It should also be noted that the strength of the bonds is also influenced by the presence of H-passivation of the oxygen dangling bonds, which further weakens the interaction between graphene and SiO_2_. The presence of strong C–O bonds would also be at odds with the values of charge carrier mobility of graphene on SiO_2_ commonly found in the literature.

In conclusion, neither the reported Raman measurements nor the cited theoretical studies provide convincing proof for the authors' claim of strong covalent C–O bonds being responsible for the observed behaviour in the piezoresponse force microscopy measurements, casting severe doubt on the schematic illustration in Fig. 6 of ref. 1, on which most of the discussion is based. As such, we encourage the authors to reconsider their argument of strong substrate-induced piezoelectricity in graphene caused by the formation of polar C–O bonds, for which there appears to be no conclusive experimental evidence.

## Additional information

**How to cite this article:** Stampfer, C. & Reichardt, S. Correspondence: On the nature of strong piezoelectricity in graphene on SiO_2_. *Nat. Commun.* 7:11570 doi: 10.1038/ncomms11570 (2016).
